# Medical History from the Earliest Times

**Published:** 1892-10-29

**Authors:** E. T. Withington


### Medical History from the Earliest Times.

By E. T. Withington, M.A., M.B. Oxon.

### XXIX.—ARABIC MEDICINE. (2) THE EASTERN CALIPHATE.

Sinan ben Tsabet (died 942), whose biography has been written by his son, Tsabet ben Sinan, forms a convenient link between the Christian and Moslem representatives of Arabic medicine. He was born a Christian, but his father having been twice excommunicated for heresy, was perhaps imperfectly grounded in the faith, and he yielded, after some resistance, to the threats or arguments of the Caliph Kaher. His fame as a physician was so great that, on the occasion of an epidemic, the Vizier, Ali ben Issa, appointed him chief of a commission for providing medical aid in the towns and villages round Bagdad. We still possess the letter of instruction in which that liberal-minded statesman declares that not even Jews are to be refused admission to the hospitals. Nay, the physician should also tend sick animals, always observing the order "first believers, then infidels; first men, and then animals." But Sinan's chief claim to remembrance lies in the fact that he conducted the earliest known medical examination for a licence to practise. In the year 931 a patient died through the fault of his physician, and all the practitioners in and around Bagdad, except those attached to the palace or celebrated for their skill, were ordered to present themselves to ben Tsabet for examination. Among the 860 who appeared was an old man, well-dressed, and of an intellectual countenance. Unwilling to question so venerable a candidate, Sinan suggested that he should favour him with a brief medical discourse. Then the old man pulled a purse of gold from his sleeve and laid it before the astonished examiner. He knew nothing, he said, of medical theories, but had long supported his family by the practice of the art, and hoped to be allowed to continue doing so. Sinan smiled, and promised him his licence on condition that he only undertook cases he perfectly understood, and never employed bleeding or purgatives, unless obviously called for. The candidate replied that such had been his invariable practice, and that he never gave other medicines than oxymel and juleps.

Among those exempted from this examination may have been a blind physician, who died full of years and honour shortly afterwards. This was Abu Bekr Mohammed ben Zechariah, called Rhazes from his birth-place, Rai, the first and most original of the great Moslem physicians (850-932.) In his youth he had devoted himself to music, but wishing for some more useful occupation, embraced the profession of medicine, and practised it with a liberality to his poorer patients which, in spite of his fame, kept him in comparative poverty, and with a boldness and originality which gained him the title of "the Experimentator."

He is probably best known as the author of the oldest existing treatise on small-pox and measles, but this work is readily accessible in Dr. Greenhill's translation, and our space may be better occupied with other subjects. Rhazes is the most independent and therefore the most interesting of the Arabic writers on medicine. Here are a few cases from the third book of his "Aphorisms" which tend to justify the title above mentioned. "A young fellow who was with me at Jerusalem complained of palpitation, melancholy, and causeless fears. After trying many things, I told him to eat hawk's flesh flavoured with marjoram and cloves, to drink white wine instead of water, and to inhale aromatic odours. Thereby he acquired fortitude and audacity, and so I cured him." "A man travelling on a hot day fell into an acute fever; his face was red, his breath hot like fire, and his heart beat violently. I waited an hour or two expecting to see some flow of blood, but nothing happened; so I ordered his nose to be rubbed vigorously. Still there was no bleeding, and the fever and pain increased. Then I gave him ten pounds of cold water to drink, and this was soon followed by copious diuresis and decrease of the fever. But his servant, who got no water because all were busy with his master, died before evening." These and other cases given by Rhazes form the nearest approach to clinical histories to be found from the time of Galen to the "consilia" of the 14th century.

Rhazes' greatest work is the "Hawi" or "Continens," which exceeds in bulk the "Canon" of Avicenna, and is written in the typical Arab style, each section beginning with a long list of authorities, "A said," "B said," "C found," &c., and sometimes ending with a modest "I say," or "I have found." We have already given a quotation from this work which, in 1395, formed the most valuable of the nine volumes, composing the whole library of the medical faculty of Paris. Shorter and better arranged is the "Liber Almansoris," dedicated to al-Mansur, viceroy of Chorasan, which contains ten books, (1) on anatomy, (2) on temperaments and physiognomy, with a chapter on slave buying; (cap. xx is short enough to quote entire, "On the significance of the ears. A man with large ears is stupid, but long lived.") (3) On diet and drugs, (4) hygiene, (5) cosmetics, (6) medicine for travellers, (7) surgical, (8) bites of venomous beasts, (9) on diseases and their treatment taken in order from top to toe—this is the famous "Nonus Almansoris," the most popular medical compendium of the later middle ages, (10) fevers. To Rhazes belongs the honour of introducing the more extensive use of chemical remedies, such as mercurial ointments; of first clearly describing spina ventosa, as well as the two diseases already mentioned; and of many novel suggestions as to treatment, of which perhaps the most extraordinary is the recommendation of the game of chess as a cure for melancholia.

Contemporary with Rhazes there lived in Egypt a physician notable both on his own account, and as the representative of his nation. The Jews, whether under Arab or Christian rule, became during the ninth and following centuries very prominent in medicine, the only higher profession not entirely closed to them, and their ablest representative was Isaac ben Solomon, called Isaac Judæus or Israeli (850-950). His fame as a practitioner spread through North Africa, insomuch that the Emir Zigadet-Allah sent him £250 and an invitation to his Court. "So I came unto him (says Isaac) and saluted him humbly, abasing myself as princes love; but I found nothing serious at his Court, but only joking and laughter." There was a certain Greek there who delighted in catching ben Solomon in logical quibbles, and obliged him once at the Emir's table to admit that "salt is sweet, and sweet salt." "But when I saw he was leading me into fallacies," continues Isaac, "I said unto him, 'You call yourself a living being?' He admitted it. 'And the dog is a living being?' 'Yes.' 'So (I concluded) you are the dog, and the dog is you.' Then Zigadet-Allah laughed aloud, whereby I saw that he cared more for wit than for wisdom." On the fall of the Aglabite dynasty Isaac returned to Kairouan, where his wisdom was appreciated, and he became tutor and physician to several Egyptian princes. Though he lived for a century he never married, declaring that so long as his work "On Fevers" survived he needed no other offspring. The book still exists, and is considered the best Arabic treatise on the subject. Among his other writings the chief is that on "Diet," a subject which he treats at greater length than any of his predecessors, even discussing the correct heat of ovens for bread-baking. But the reader may be more interested in the Jewish physician's estimate of pork. "The flesh of pigs is most nutritious; it forms a good chyme, and by its humidity and viscosity preserves the moisture of the stomach. It is diuretic and not suited to those who eat little. It is useful for those of hot and dry temperaments, for they readily digest and thrive upon it; but for those of an opposite complexion and of weak digestion, it is noxious, generating evil humours and a viscid phlegm, which may produce gout, lumbago, sciatica, renal stone, and paralysis. The flesh of old and decrepit pigs is most unwholesome; it is hard, woody, and insipid; those who habitually eat it fall into melancholy and hectic fevers." The above works were written in Arabic, but ben Solomon has also handed down his wisdom in "The Physician's Guide," a Hebrew book of proverbs or aphorisms, of which the following are examples: "When a patient can be cured by diet use no drug, and when simple medicines suffice avoid complicated ones." This is also given by Rhazes, and may have been copied from him. "Treating the sick is like boring holes in pearls, and the physician must act with caution lest he destroy the jewel committed to his charge." "Make your fees as high as possible, for services which cost little are little valued."

